# The Present State of Microscopical Science, Medically Considered

**Published:** 1859-07

**Authors:** John H. Packard

**Affiliations:** Philadelphia


ART. II.—*The Present State of Microscopical Science, Medically Considered.* By JOHN H. PACKARD, M.D., of Philadelphia.

(CONTINUED FROM MAY NUMBER.)

THE previous portion of this article having been devoted to the discussion of the cell and its development in the several tissues, we are ready to take up

III. *Such processes as are open to microscopical study, and yet involve several textures.* Of these the most important is the capillary circulation, which can readily be seen in the web of the frog's foot, in the tail of the tadpole, in the wing of the bat, and in the lungs and mesentery of small animals. In health, a continuous current rushes through the capillaries, the red disks moving rapidly along the centre of the channel, and the white corpuscles forming the so-called "still layer" at the periphery. This still layer does not exist in the lungs, at least in the capillary rete connecting the pulmonary arteries and veins; for here the main object to be attained is the aëration of the red corpuscles. But here, as elsewhere, whatever interchanges may go on between the air or the tissues and the blood-corpuscles, the liquor sanguinis probably acts as the medium of communication.

From the continuous character of the current seen in the capillary vessels under the microscope, must be inferred the necessity of some other agent than the heart for carrying on the circulation; and thus we are compelled to recognize an affinity existing between the tissues and such red corpuscles as have not yet lost their oxygen. The idea of such an affinity not only serves to explain some points in connection with secretion and growth, but has an important bearing upon the general theory of life. Each ultimate structural element may be said to possess a certain power over the blood, an elective affinity for just those substances which are needed for its nutrition or for the exercise of its function. Nor is it more difficult to conceive of such a special power of attraction, than to assign any other rationale for the distribution of the various secretions.

Having placed in view the capillary circulation of the web of a frog's foot, we may, by applying a local irritant to any point in it, induce a condition of the vessels at that point imitating more or less closely that which occurs in inflammation.

Two chief phenomena ensue when this is done: in the first place, the vessels alter their calibre; secondly, they become crowded with red corpuscles.

Concerning the former phenomenon, there has been much discussion; some asserting that there is first a contraction and then a dilatation, others, that the dilatation is primary; the latter party are again at variance as to the action or passive character of the enlargement. Some observations published by Mr. Lister* bear upon these points.

Mr. Lister states that in the minute arteries of the frog's foot he has detected muscular fibrils of the non-striated variety, winding spirally between the inner and outer coats; a circumstance clearly explaining the occasional occurrence of contraction as a primary effect of irritation. And as the contractility of the fibres must necessarily be at length exhausted, we have one reason why dilatation should follow after a time.

But the nutrition of the part is under the influence of the nerves with which it is supplied; and no irritation can be so managed as not to affect their terminal fibrils. Here then, in the nervous excitement, giving rise to an increased demand for blood-corpuscles, is another cause for the dilatation of the vessels. We think it may be asserted that, except when the non-striated muscular fibres before alluded to are thrown into action, the walls of the vessels are perfectly passive, dilating simply because of the mass of corpuscles *drawn into them by the surrounding tissues.*

And this leads us to the second phenomenon, the crowding of the capillaries with red corpuscles. The tissues, having attracted an excess of corpuscles, are so deranged functionally, that they do not, as usual, still demand more, and thus keep up the succession; hence there is actually no force to move the mass along. All around the focus where this takes place, not only are the corpuscles moving which would properly have traversed the obstructed channel, but a minor degree of the same excess in the attractive power prevails. The blood-corpuscles, thus crowded together and stagnant, break down; their coloring matter passes into the liquor sanguinis, which oozes through the walls of the vessels and stains the surrounding tissues.

The products of inflammation, varying in different cases, are of interest when studied microscopically. Of these, we recognize only *lymph* and *pus;* for we should not speak of a merely increased secretion, or of blood, as a product of inflammation.

Lymph is found under the microscope to consist of corpuscles, very similar to the white corpuscles of the blood, floating in a clear fluid. And wherever poured out, it has these anatomical elements; no difference exists between that found upon a mucous and that upon a serous surface, although incidental circumstances modify the results in each case. As has been previously remarked, there is a very intimate relation between mucous and serous membranes; and the lymph-corpuscle which in one, placed under conditions unfavorable to its higher development, becomes a

* Transactions of the Royal Society of Edinburgh, vol. xxi. p. 549.

pus-corpuscle, will in the other go on to the formation of a perfect fibre. The greater activity of cell-production in the mucous membrane, and the less persistent character of the cells, with the constant escape through them of certain matters from the blood, would lead us to expect just what takes place. Lymph poured out on the surface of the pericardium or pleura, is much more under the assimilative influence of the tissues than that effused in the vagina or urethra; but let the distinctive incidental circumstances be changed,—let the pleura be opened to the atmosphere, or two mucous surfaces less protected by epithelium than usual be placed in contact,—the results are changed also.

The process of suppuration is in some respects quite analogous to that of secretion, with which it was formerly considered to be identical. Plastic matter is poured out from the capillary network underlying a surface, and is by its very nature disposed to assume a form; but, little or no assimilative force being exerted over it, it fails to acquire any but the very lowest type of organization. Some pathologists regard pus as degenerated lymph; but if it is so, the conversion of the lymph-cell into the pus-corpuscle must occur almost at the very moment of its formation. Many circumstances go to prove an extremely intimate alliance between the two substances; but the precise relation between them is still undecided, and seems indeed to vary in different cases. Thus in purulent ophthalmia, the exuded material presents the characters of pus *ab initio;* in empyema, and in acute abscesses, the breaking down of effused lymph undoubtedly takes place.

Microscopic researches have shed much light upon the mode in which injuries of the different tissues are repaired. The material poured out from the blood-vessels for this purpose assumes the form of nucleated cells or nucleated blastema, and is subsequently influenced very much by the surrounding tissues, coming at last to resemble them to a greater or less degree. But the point most discussed in connection with this subject is the manner in which the new blood-vessels are formed; whether the loops or arcades of capillaries, discernible in thin perpendicular sections of granulating parts, are outgrowths from the vessels below, passages channeled by blood-corpuscles forced through the organizing mass, or the result of an inherent power in that mass to form its own vessels and its own circulating fluid. This latter view is sustained by some undoubted facts, but only in exceptional cases. Generally, pouches or cul-de-sacs sprout out from the neighboring capillaries into the reparative material, and by their meeting and coalescence form arched tubes; according to Mr. Paget,* if one of these pouches should burst, the blood-corpuscles, thus

* Lectures on Surgical Pathology, Am. edition, p. 147.

set free, will still go on in the proper direction, and the final result will be the same.

Besides the processes now enumerated, there are two others, which, although properly claiming earlier notice, may be most conveniently spoken of in this connection.

*Muscular contraction* has been already alluded to in a former part of this article. It may be observed by placing under the microscope a few fibres cut from the leg of a living frog, and irritating them by means of a hair introduced beneath the thin glass cover; the effect is better if strychnia is previously administered to the frog. From some experiments made by Dr. C. S. Boker and myself, it appeared that a galvanic current passed through an isolated muscular fibre was entirely without effect. We also found that contractions sometimes took place in the individual fibrils composing the fibres, so that the striæ, instead of running directly across from side to side, became zigzagged; a small part only of any one stripe was involved, but the adjacent ones were affected likewise, so that rows of the ultimate sarcous elements were seen to act in concert.

Another very interesting phenomenon, not discoverable without the aid of the microscope, is *ciliary motion*. This may be best observed in the ciliæ of the epithelium from the trachea of a newly-killed animal; it seems to be independent of any muscular action or other mechanical agency, and ceases spontaneously after a certain length of time; it may, however, be re-excited by the application of a solution of potassa. Its object is supposed to be the propulsion of particles of mucus, etc. toward different outlets; and I believe it does not occur where this function can be fulfilled by muscle.

IV. *Such structural elements as constitute morbid deposits.* This subject is involved at the present day in peculiar uncertainty and confusion, and hence must be discussed with as much circumspection and impartiality as possible. And here we may at once admit that the revelations of the microscope have given rise to much of this obscurity; but so far from regarding such an admission as casting any discredit upon the instrument, we shall try to show that the truth of the whole matter is most likely to be arrived at by its aid. Dealing with material and visible phenomena, we must hold theory and hypothesis in abeyance, trusting to patient research and close deduction, certain that in this way alone can nature be made to disclose her workings. To give up in despair, or to jump at a conclusion from insufficient data, would be equally unworthy of a scientific mind.

Our present task is simply to survey the field of labor, in order to note what is established and what doubtful; endeavoring to introduce into the discussion as little irrelevant matter as possible.

A morbid deposit may be supposed to consist either in an undue increase in the quantity of any tissue, in the occurrence of any normal tissue in an abnormal situation, or in the organization of blastema according to a type differing from that of any normal structure. Under the first and second of these heads would be included the homologous growths; the third would comprise the heterologous. As long as the general shape and structure of the organ in which the deposit occurs is not materially altered, the term hypertrophy is used to express the condition of things, and with this we have now nothing to do. But when the new material is thrown out as it were in a purposeless and irregular manner, it forms a *tumor*, be it like or unlike any healthy tissue—homologous or heterologous.

In the earlier days of microscopical science, a visible difference was asserted to exist between the ultimate structural elements of homologous or benignant, and heterologous or malignant tumors. And many writers still employ expressions indicative of a belief in such a difference; we read of cancer-cells, the microscopic characters of cancer, etc. But no one can become practically acquainted with this subject without finding that malignancy adheres to no special morphological type. In other words, a microscopic examination, made without reference to clinical history, cannot in all cases be depended upon.

A doctrine which finds great favor at the present day, and of which Virchow and Wedl are the chief exponents, may be briefly stated as follows: The structural elements of all new growths have the fundamental characters of normal cells, disguised variously by incidental influences; malignancy is merely relative, and depends upon some *dyscrasis* of the blood not yet elucidated.

Much may be said both for and against this view. It has the advantage of simplicity, and seems to narrow down the unexplained part of the subject; but at the same time it transfers the enemy to a more impregnable position. Several facts seems to bear it out by analogy; thus the pus of gonorrhœa, absolutely identical in physical characters with that from an ordinary abscess, has yet an undoubtedly specific nature. The arguments against it are mainly derived from clinical observation, the general course and symptoms varying so widely and so constantly in cases of cancer or tubercle from those in cases of innocent tumors.

Having now very briefly stated the main points of the great vexed question in pathology, let us take a concise survey of morbid deposits in their microscopical aspect; arranging them altogether with a view to convenience.

*Fatty* tumors, whether continuous or not with the normal *tela adiposa*, are the simplest of all out-growths, and possess no interest except from this fact.

*Fibrous* tissue, and its allied forms, constitute a very extensive and important variety of morbid deposit. Many growths of this kind, seemingly of very diverse character, are shown by the microscope to be anatomically identical, the differences between them residing in the arrangement or in the stages of development of the tissue in the several cases. Polypi, fibro-cellular and fibrous tumors, and those known as fibroid, are thus placed in the same category; the "painful subcutaneous tumor" is often, though not always, to be added to the list.

Now, in nearly all confessedly malignant growths, fibrous tissue is found in more or less quantity, and in all stages of development; in some cases, indeed, this seems to be the only element concerned. Polypi, especially in the antrum maxillare, are well known to be suspicious in their character, and sometimes unequivocal. Tumors consisting apparently of the purest fibrous tissue occasionally exhibit such a clinical history as places their malignancy beyond a doubt; and the same may be said of the fibroid and fibro-nucleated growths so well described by Paget. In view of these facts, we cannot assign the family of fibrous or fibroid tumors a place among homologous or benign growths; their position is a doubtful one, and must remain so until they have been further studied.

*Cartilage*, *fibro-cartilage*, and *bone* may occur as morbid deposits. Either may exist by itself, or they may be variously combined; and the degree of structural perfection attained varies in different cases. Perhaps no class of tumors presents so great a variety of microscopic appearances as those composed of cartilage and fibro-cartilage, in proportion to the number of cases met with; and these growths can hardly be assigned a definite position as yet. They sometimes present clinical phenomena savoring strongly of malignancy, but are in general perfectly benign.

When bone is formed, it may be developed through cartilage, but more frequently through fibrous tissue; and here also every gradation is observable, from a mere stelliform deposition of earthy salts to the complete Haversian ossicle. Nor does any assimilative force seem necessary to initiate the action, since Dr. Ogle* found canaliculi and lacunæ in a phlebolithe, and osseous formations are not uncommon in the membranes of the brain and spinal cord; they also occur in connection with malignant disease of the testis. Microscopic examinations are of great value in the study of these deposits, especially when aided by chemical tests. Thus the so-called ossification of arteries may be sometimes shown to be a mere atheromatous degeneration, and to depend for its hardness upon the solidification of cholesterine.

---

* Trans. of London Path. Society, vol. vii. p. 133.

Another form of tumor would seem to be mentioned most appropriately here,—the *myeloid;* since, as its name imports, it presents a structural analogy with the medullary tissue of bone. Generally found in connection with bone, these growths sometimes become to a greater or less extent ossified; thus bearing out the idea of an intimate alliance between them and the normal texture alluded to. But they, like several of the preceding forms, sometimes run a course indicating malignancy; so that of them also it must be said that their true position, if constant, cannot at present be assigned.

*Muscular* tissue of the unstriped variety is met with in fibrous tumors of the uterus, where its presence may be readily accounted for. No instance is upon record in which striated muscle has been found as a mere outgrowth, with or without other tissues.

*Nerve*-elements are present in some tumors, but the rank they assume is doubtful. Thus it may be questioned whether they belong to the new growth, or are merely surrounded by it—carried away as it were by the force of external circumstances; which latter seems most probable in the majority of cases. In the "painful subcutaneous tumor," the excessive tenderness is not owing to nerve-structure entering into the new growth, but merely to the intimate connection of the latter with some nerve-fibre, which may be in an entirely normal state. And even in the so-termed *neuroma,* the proper nerve-substance is not increased, but simply the fibrous tissue of its investing membrane.

*Glandular* tumors have been made the subject of careful study by many pathologists. The most familiar instance of them is, perhaps, that called by Lebert "partial hypertrophy" of the mammary glands. As their name implies, they consist of the elements of gland-tissue, irregularly placed.

Now it seems to me that the scope of this name should properly be wider than it is. It has several times occurred to me to detect a regular epithelial lining in cysts connected with the ovary, and between these and the epitheliomata there seems to be the same analogy as between serous and mucous membranes. Wherever they may occur, morbid deposits are, as has been mentioned, more or less under the influence of the laws governing healthy nutrition; and therefore they should certainly be grouped for study according to the normal elements with which they range themselves. Hence, if the histological views expressed in a former portion of this article be correct, all morbid growths connected with serous and mucous membranes, and with glands generally, should be placed in the same category.

Tumors of this kind, as might *a priori* be expected, present every stage of complexity of structure; from the mere aggregation of epithelial cells, some epitheliomata advance to an arrangement of them in concentric layers; fibrous tissue in variable quantity may be added to this; and so by successive steps the tubular ramifications, lined with epithelium and surrounded by a stroma of connective tissue, are arrived at, and we have the acknowledged "glandular hypertrophy."

The occasional deposit of cholesterine in the crystalline form among the cells making up an epithelioma, (constituting the *cholesteatoma* of Müller,) must be regarded as akin to similar deposits in cysts which have undergone inflammation, and affords another proof of the alliance between the two forms of disease. In the cyst the fundamental gland-structure is in a state of involution, in the epithelioma it is in a state of evolution.

Concerning that anomalous substance, *colloid* matter, very little need be said here. Its presence does not imply a malignant character in the growth which contains it, although it forms a very large proportion of the mass of some cancerous tumors; its position structurally is extremely low, and not very well defined.

In the course of the foregoing discussion allusion has of necessity been made to malignancy, as a character often assignable to outgrowths; and it has been stated that this may be manifested by some tumors, whose form-elements do not differ from those of such as are quite innocent. Such cases, instead of affording evidence against the value of the microscope, simply show its true office as an auxiliary to clinical observation; that the field of its operations is even wider than its earlier advocates asserted.

*Tubercle*, which from its relations to the system at large, and the evident tendency it so often displays to spread among the tissues adjacent to it, seems to merit the epithet "malignant," has microscopic characters by which it may, in general, be readily recognized. Lymph or pus, in a state of degeneration, may have somewhat the same appearance; and hence caution should be observed in cases where any such error might arise.

Lebert's description* of the "tubercle-corpuscle" is so clear and excellent, and sets forth so well the views commonly held upon this matter, that I subjoin a translation of it. After speaking of the molecules and yellowish intercellular substance met with in tubercle, he says:—

"(3) *Corpuscles or globules peculiar to tubercle.*—The constant and characteristic element of tubercle is the tuberculous globule, (*globule tuberculeux,*) which, as we shall presently see, is distinct from every other normal or pathological primitive element. It is rarely round or oval; but, although irregular in shape, it always approaches more or less

* Anatomie Pathologique, vol. i. p. 332.

one of these forms. Its outline is usually angular, rounded when seen only from one side, but rather polyhedral when floating freely, a condition necessary for the accurate observation of its surface. This latter, although irregular, is smooth, and has no granular matter adhering to it. The volume of these globules varies between $\frac{1}{400}$ and $\frac{1}{120}$ of a millimètre; we have sometimes seen it as much as the $\frac{1}{100}$. These measurements are the mean result of a great many observations. When the globules are more ovoid, the mean of their width is $\frac{1}{130}$ of a millimètre, while their length varies between $\frac{1}{125}$ and $\frac{1}{160}$. The contents of these globules consist in a more or less transparent matter, with molecular granules. We regard the former as somewhat tenacious, (*solide,*) having never observed within these corpuscles the molecular movement visible when many granules exist in a liquid medium within the cell.

"The contained matter is sometimes granular; sometimes again there exists a sort of lacuna more transparent than the rest. Real nuclei are rarely seen here. In an exceptional case of tubercle in the vertebræ, the ordinary tubercle-corpuscles, irregular in shape, contained a nucleus $\frac{1}{200}$ of a millimètre in diameter, with one or two very minute nucleoli. This important fact argues in favor of an opinion we long since advanced, viz., that the peculiar corpuscles of tubercle are imperfectly developed cells; it is likely that the firm consistence of the surrounding blastema hinders their evolution. Hence we now and then observe in tubercle cells more fully completed. It seems as if the blastema became firm so quickly as to prevent the differentiation of the cells into cell-wall, nucleus, and nucleolus. If we claim for tubercle a special element of diagnostic value, an element of which the shape and appearance are mainly due to the conditions of its development, we would not pretend that herein resides the peculiar nature of tubercle. Time alone will show whether such elements belong to tubercle, cancer, etc. For the present, we cannot but insist upon the special form of these elements, without trying to determine their precise value and bearing. The granules inclosed within these corpuscles vary in number; sometimes there are four or five, sometimes ten or more. They are never so numerous, however, as in the granular corpuscles, properly so-called; a few of them only can be seen at once, because the globules are almost as deep as they are long and wide, and hence can only be in part exactly in focus, when viewed with high magnifying powers. The transparent granules within them have not the aspect of nucleoli. The color of tubercle-corpuscles is a pale yellow, but is entirely lost when they are highly magnified."

*Cancer* has, for obvious reasons, constituted one of the most interesting of all the subjects embraced in microscopical pathology. Owing to the rash statements and hasty conclusions of some of the earlier micrographers, the question whether any particular growth did or did not partake of this nature, was for some time expected to receive a positive solution as the result of microscopic study alone; and this too from a mere glance, perhaps, at an insignificant scrap cut from any portion of the specimen. Further experience showed the fallacy of this expectation, the anatomical elements of tumors, undoubtedly cancerous, being found far from uniform; and now the favorable opinion formed of the microscope, based originally upon insufficient evidence, was abandoned with equal or even greater readiness. But there were not wanting those who had from the first observed a prudent caution in the matter, and who still adhered to their philosophical pursuit of the truth. Among these must certainly be numbered the German school, represented so ably by Virchow and Wedl; the views of these pathologists have already been alluded to, and need only be mentioned again for the sake of a respectful expression of dissent. Neither in France, England, nor America, have these views been generally received as yet, mainly, I think, because they sustain a less satisfactory theory than that which would seem to be unavoidably abandoned in accepting them. For if the material by which cancerous disease is represented in the economy be *nothing but* areolar or connective tissue, or epithelium,—like a body of well-meaning but illiterate men seduced into acting as tools in a bad cause,—then either there is no such thing as cancer, (in which case the general rejection of Bishop Berkley's opinions might as well be reconsidered,) or else the whole matter is made more incomprehensible than ever. Nor does there seem to be any reason for denying a specific character to the cancer-cell, even if the peculiarity be invisible; certainly the pus of a chancre, or of gonorrhœa, is specific, although to all appearance identical with that of any ordinary abscess. But more than this, we do find in every normal texture of the body a certain type, pretty closely adhered to by all the constituent anatomical elements. In cancer, on the contrary, it is common to meet with a very great variety of cell-characters.

As far as I can ascertain, the opinion of most American pathologists coincides with that of Lebert, whose view of the "cancer-cell" is as follows:—*

"The type of the cancer-cell is a small regular sphere, with an elliptical nucleus eccentrically placed, occupying nearly half, or more than half of the cell-cavity, and inclosing one or several large nucleoli. But this type is not often met with in perfection, (*bien pur;*) the cell-wall assumes an ovoid or elongated form, or becomes triangular, with angles either acute or obtuse, or fusiform, pointed at each end and wide at the middle; or again

* Anat. Pathologique, vol. i. p. 279.

it may present, in place of two appendages opposite to one another, (*verticaux,*) a single long and pointed appendage, passing off from a round cell, or three or four, likewise long and pointed, passing off from an ovoid or irregular cell. It would be useless and even impossible to describe all these forms here; it is enough to mention the fact, that this multiformity of the cell-wall is not observable in an equal degree in any other species of cell."

Among English writers, Mr. Paget has perhaps discussed this subject more fully, in the aspect which now concerns us, than any one else; and from the tone usually assumed by his countrymen, in connection with reports of cases, (a criterion quite as accurate, I take it, as any formal statement of opinion would be,) his views have met with their very general approbation. These views seem to me to be set forth in the following passage from the valuable work already several times referred to:—* "Together with the disorderly construction, and the peculiar cell-forms, we may often observe, as characteristic of cancers, the multiformity of the structures composing their mass. It is not equaled, I think, by any tumors, unless they be the cartilaginous, or the mixed glandular and cartilaginous. * * * It would be too tedious even to enumerate more forms than these of the component cancer-structures, and I need not again describe them. It is not their multiformity, so much as the existence of many of them in a single mass, that is generally characteristic of cancer.

"Various as are these corpuscles of cancers, it is yet to be observed that there are none so entirely different from those of normal structures that we cannot point out among them their type or parallel. No observation since Müller's time has invalidated his demonstration of this principle. The experienced microscopist will, indeed, very rarely fail in the diagnosis of a cancer by its minute structures; but he only discriminates them as specific modifications of the nucleus, nucleated cell, endogenous cells, and other forms, of which the types are in natural parts; he finds among them no new type-forms."

I think it may safely be said that these ideas of Mr. Paget's are in accordance with those of a large majority of the microscopists of this country; although the discussion of the subject is at present so incomplete, and is attracting so much attention, that hardly any surmise can be attempted as to its event. Perhaps no other question in pathology could have been debated, as this was a few years since, in the *Academie de Mêdecine* in Paris, with so unsatisfactory a result.

From what has been stated under the present head, it is manifest that

---

* Lectures on Surgical Pathology, Am. ed., pp. 654–55.

the opinions of the profession in regard to the pathology of tumors are far from settled. But it does not follow that they never will be. On the contrary, the earnest scrutiny which all observations and theories bearing upon this very wide and important subject are now undergoing, warrant the hope that further light may be obtained. Let it be remembered that as yet we are hardly beyond the threshold of this department of inquiry; that no real advance can be made except by the impartial as well as critical examination of all the available facts; that prejudices and pet theories may have to be sacrificed, but at last we shall arrive at as much of the TRUTH as is granted to finite minds to know. Here is the great aim of science, and to this end all her votaries must use their powers, in whatever branch of her service they may be engaged.

V. *Matters secreted and excreted.* Under this head we have to deal with substances separated from the blood, either by way of mere elimination or for the fulfilment of some further purpose. These substances are produced as the result of the life-actions, healthy or morbid, of different organs; and when examined microscopically, some of them display characters allied to those of the living cell, while others are in the form of mere crystals. But they are all, practically speaking, without the sphere of vitality; none of them, so far as we know, are organizable.

Of the forms allied to living cells, the *spermatozoid* is doubtless the most important. The value of its microscopic recognition in cysts connected with the testicle, in the urine, and in cases of alleged rape, need not be enlarged upon. As yet, the mode in which its fecundating influence is exercised upon the ovum has not been determined; in some of the lower animals it has been shown to penetrate the ovum, and then to disappear, but this process is not known to occur in mammalia.

The *ovum* itself is in a manner secreted within the cavity of the Graafian vesicle; its cell-nature has been already mentioned, and the analogy of the vesicle with other glands is quite as clear as that of the solitary and agminated glands of the intestine, since it, like them, discharges its contents by dehiscence upon the mucous surface of a canal leading outward.

*Mucus*, wherever found, has certain distinguishing corpuscles, pretty closely resembling those of lymph, and somewhat also those of pus. *Saliva* from the front part of the mouth is merely a dilute mucus, whose corpuscles are somewhat more granular, and which contains also epithelial cells; that derived from the tonsils and fauces differs from this only in being less dilute.

*Milk* is characterized microscopically by a great abundance of oil-globules, which are perhaps true Aschersonian bodies, having only a caseous instead of an albuminous envelop. In the colostrum-corpuscles, usually seen in the milk of the first few days, it has occurred to me to detect nuclei with nucleoli; this appearance has indeed been uniformly present in the specimens I have examined, although it is spoken of by most authors as exceptional, if alluded to at all. Reinhardt's theory, that these bodies are the effete epithelial cells of the lactiferous tubes, does not seem to be favorably received by physiologists, and their office remains unexplained.

The *bile* has been carefully studied by some microscopists; the crystals thus observed in animals are mainly acicular, variously grouped, and composed of glyko-cholate of soda, the tauro-cholate of soda being uncrystallizable. Cholesterine is discharged from the blood by way of the liver, but is not found crystallized in the bile. Human bile contains no crystals or solid elements of any kind, and is therefore without the province of microscopy.

The *urine* has received very great attention from many microscopic observers, especially on account of the clinical advantages derivable from this source. Of the various deposits which occur in morbid urine, some indicate disease of the kidney, others alterations in the mucous membrane of the bladder; others again are significant of pathological changes in more or less remote organs.

So general is the recognition of the usefulness and authority of the microscopic study of these substances, that any extended discussion of the matter would be unnecessary here; nor indeed could any other than a very imperfect sketch be given in the space at command. But perhaps an enumeration of such urinary deposits as have hitherto been subjected to this method of examination may aid our present purpose.

From healthy urine there may be obtained—

Urea;
Uric acid;
Creatine;
Creatinine;
Hippuric acid;
Chloride of sodium;
Phosphate of lime;
Crystals accidentally present, derived from the food;
Epithelial and mucus-corpuscles.

Pathological urinary deposits have been variously classified by different authors. Some of them are derived from the metamorphosis of the tissues, some from that of the food, and some are merely inorganic matters, which have never actually become part and parcel of the economy. Laying aside, however, all reference to the mode of origin of the solid matters found in morbid urine, as irrelevant, we may arrange them into such as have a crystalline form, and such as have an organic form.

Among the crystals, there are found—

- Uric acid;
- Urates of { soda, ammonia, magnesia;
- Hippuric acid;
- Oxalate of lime;
- Purpurine;
- Cystine;
- Phosphates of { lime, ammonia, magnesia, } Ammonio-magnesian phosphate;
- Carbonate of lime.

Among the organic forms, (parasites being alluded to hereafter,) we have—

| | |
|---|---|
| Blood, | Pus, |
| Mucus, | Epithelium, |
| Spermatozoids, | Torula cerevisiæ,* |
| Milk, | Oil-globules. |

In the study of many of the crystals above named, the employment of polarized light not only produces the most beautiful appearances, but is of essential service in distinguishing between one substance and another.

*Fæcal matter* has been investigated microscopically by Frerichs,† Hammond,‡ Höfle, Wehsarg,§ Ihring,|| and others. The organic constituents thus detected are partly the remains of substances taken as food, such as muscular fibre, more or less broken up, fibrous tissue, fatty matters, the indigestible portions of vegetables, and sometimes starch-corpuscles; epithelial cells and mucus-corpuscles from the lining membrane of the intestine, and occasionally, in diseased conditions, blood-disks. The most frequent inorganic constituent is the ammonio-magnesian phosphate; Marcet¶ found also a peculiar substance, whose crystalline form was an

---

* In diabetes. Dr. W. A. Hammond has also found it as a result of an exclusively amylaceous diet. (*Prize Essay of Am. Med. Assoc.*, 1857.)

† Wagner's *Handwörterbuch der Physiologie*, art. *Verdauung*.

‡ Prize Essay of Am. Med. Association for 1857.

§ *Mikroscopische und Chemische Untersuchungen der Fæces gesunder, erwachsener Menschen.* Giessen, 1853.

|| *Mikroscopisch-Chemische Untersuch. Menschl. Fæces unter verschiedenen pathologischen Verhältnissen.* Giessen, 1852.

¶ Philos. Trans., 1854.

acicular four-sided prism, and to which he gave the name of *excretine.* Perhaps the further pursuit of these investigations may shed additional light upon the later stages of the process of digestion.

VI. *Animal and vegetable parasites.* With regard to some of these organisms there is room for doubt, whether they should be looked upon as the causes, the effects, or the concomitants of disease. They have been studied by many distinguished observers, among whom may be mentioned Diesing, Küchenmeister, Robin, and Leidy, as perhaps the most authoritative. We have, of course, to speak here of those parasites alone whose existence is revealed by means of the microscope; although this instrument has afforded very valuable information concerning the anatomy and physiology of those large enough to be detected with the naked eye.

Some of these parasites exist within the sphere of life; thus the *Trichina spiralis* is found embedded within the voluntary muscles. Valentin* has described a parasite, large numbers of which were seen by him in the circulating blood of the *salmo fario,* (salmon-trout;) it consisted of a single cell, with processes extending from it in different directions. A similar organism was seen by Gluge† in the blood of a frog; and it is suggested by this observer that a connection may perhaps be found to exist between these parasites and intestinal worms.

So late as 1834, M. Breschet read before the Academy of Medicine in Paris, a paper, in which the idea was seriously entertained, that pulmonary tubercles may be of parasitic origin; but no sufficient grounds exist for adopting such a view.

All true parasites, whether animal or vegetable, are derived from sources external to the body; and with the exception of those already mentioned, they are all found upon the surface-layer, cutaneous or mucous.

Upon the skin of man there are only two microscopic animal parasites now known, viz.: the *demodex folliculorum,* and the *acarus scabiei* or *sarcoptes hominis.* The vegetable formations are more numerous; an alga called the *leptomitus epidermidis* has been observed in vesicles which appeared after the application of a poultice, and fungi of various kinds are found in ulcers, in chloasma or pityriasis versicolor, in herpes tonsurans, in mentagra, in plica polonica, in porrigo, and in favus. I have myself met with wisp-like formations of fine fibres in the vesicles of herpes phlyctenodes and herpes zoster, and have found in eczematous scabs epithelial scales crusted over with spores; sometimes these latter were floating freely in very great numbers, tending also to arrange themselves here and there in dendritic forms. And a fungus is described by Meissner and

* Müller's Archiv., 1841, p. 435.

† Ibid., 1842, p. 148.

Virchow as occurring in the finger and toe-nails in certain morbid states.

In the mucus which collects around the teeth, upon the edges of the gums, there is found a parasitical animal consisting of a single cell, and called the *denticola hominis.* A species of alga, the *leptothrix buccalis,* inhabits the dorsum of the tongue, and the spaces between the teeth. The *leptomitus Hannoverii* occurs on the mucous membrane of the tongue and pharynx in some low forms of disease. Perhaps, however, the most important of all these parasites is the *oidium albicans,* the fungus which is found in aphthous ulcers of the mouth.

In the stomach and intestines we sometimes find the yeast-plant,—the *cryptoccus* or *torula cerevisiæ;* and under circumstances not yet clearly made out, the *sarcina* or *merismopædia ventriculi,* discovered by Goodsir in 1842; both these are algæ. A confervoid growth has been described by Dr. Farre, as detected by him in the alvine discharges of a dyspeptic patient who had an attack of colic; he gave it the name of *oscillaria intestini.*

In discharges from the urinary bladder two animal parasites are said to have been discovered: the *dactylius aculeatus* and the *spiroptera hominis,* both which are allied to the nematoid worms. Two forms of algæ are sometimes evacuated in the urine,—the *leptomitus urophilus,* and the *torula cerevisiæ* or yeast-plant,* before alluded to as occasionally met with in vomited matters.

Another species of *leptomitus* is found in the uterine mucus, and still another is described as seen in a puriform discharge from this organ. In the eye a parasite of the same variety has several times proved a source of great disturbance. The *trichomonas vaginalis* is said by Donné, Dujardin, Kölliker and others, to have been detected in unhealthy vaginal discharges.

Lastly, Bennett and others have described certain fungi as occurring in inflammation and gangrene of the lungs. Here we may readily suppose the spores of these organisms to have been taken in in the process of respiration, and their development to have been favored by the state of the diseased parts, just as when mould forms upon a decaying vegetable.

It is extremely probable that further researches may extend the list of parasitic growths, perhaps to a much greater length. Great care is, of course, necessary in pronouncing upon the nature of any organism sus-

* It is doubtful whether this organism is really formed in the bladder, or is merely a result of fermentation outside of the body. As was said in a previous note, it is met with in cases of diabetes, and also when the diet has been exclusively amylaceous, sugar being as a consequence formed in the urine. Strictly speaking, it has no claim to a place among parasites.

pected to belong in this category, on account of the unavoidable risk of error; matters whose presence is purely accidental may be assigned undue importance, while such as really claim attention may be overlooked entirely.

The foregoing survey of the ground now occupied by microscopy, although of necessity very concise, may serve to shadow forth what has already been done in this department of research, as well as in some measure also to indicate the direction in which further advances may be made.

Still another object has been indirectly aimed at,—the vindication of the microscope from the aspersions cast upon it even by some who are themselves reaping the benefit of its revelations.* Granting that mistakes have been made with it, that rash assertions and unsound arguments have been put forth as the results derived from its use, by incompetent or careless observers, there yet remains a mass of undoubtedly valuable knowledge acquired by its aid. Should the microscope be discarded on account of the errors of some of its advocates,—should truth and its pursuit be given up because falsehood and misconception have existed, man might as well abjure his intellect because it is not infallible, and seek the level of the unthinking beast.

Let it not be thought, however, that an apology is meant to be offered for the microscope. Nothing of the kind is needed. If it has been shown that positive advances have been made by means of this instru-

---

* In a recent work on physiology the following language is used:—

"*When we consider, therefore, the constant deceptions of the microscope, especially in all explorations of soft substances, and the absolute uselessness of any knowledge it may convey as to the recesses of organization, it may be reasonably expected that the time is not distant when all this lumber will be excluded from practical works on physiology, and turned, at least, into a channel by itself.*" (Paine's *Institutes of Medicine*, p. 60.)

If this author excluded from his book everything based upon microscopic observation, (as would be expected of any one entertaining such an estimate of the latter,) he could hardly have looked for his readers among the men of the present day; if he did not, there is no slight tinge of unfairness in expressions such as the above. It must surely be surprising to those conversant with the existing state of anatomical science, to find on page 129 of the work just quoted, the assertion, that "*the lacteals have open orifices in the intestinal villi.*" Nor can any one, who has ever studied the circulation microscopically, admit that there is the slightest truth in the statement made as follows, on page 485: "*Irritants of a chemical nature have been applied to delicate membranes, by which their organization is impaired or destroyed, and the blood also coagulated by direct chemical influences. The part has then been subjected to the microscope, under the direct rays of the sun, whose heat has the effect of drying the disorganized tissues, and consolidating the blood.*" How far the author of such remarks is qualified to pronounce sentence upon microscopy, I leave the reader to judge.

ment, and that the field of its usefulness is not yet cultivated to exhaustion, neither its opponents nor its advocates can demand more.

What is here claimed as an established proposition is, that the microscope merits a place among the reliable means of medical research. And just as information derived in any other mode, as for instance by observation with the naked eye, must be weighed and analyzed, so must that now in question. Just as an accumulation of facts is essential for the support of any general law in nature, so must it be in minute anatomy and pathology. Every physical law is based upon observed phenomena, error being as much as possible eliminated from such observations; and herein, to a great degree, lies the value of the inductive method of reasoning,—one fact checks another, and the greater the number of concurrent facts, the stronger the compound fact or law derived from them.

Now in studying the phenomena of life, whether in health or in disease, the conditions to be taken into account are extremely numerous, complicated, and variable; and hence, not only is the utmost possible circumspection necessary, but all available means must be brought to bear upon the subject. To overlook or set aside any method of inquiry which can be suitably employed, is simply to detract so much from the solidity of the result, whatever this may be. Wherever the testimony derived from different sources is found to be conflicting, we may be sure that the fault lies either in the incorrectness or incompleteness of our observation of facts, or in the wrong construction placed upon them.

Certain precautions on the part of microscopists and micrographers are absolutely essential, or the claims here asserted must prove untenable.

It must never be forgotten that the microscope is a mere optical instrument, capable only of presenting the image of any object, as if the latter were at a less distance than it really is from the retina. And in estimating the value of any statements based upon its testimony, we must assign it the same position that we should a pair of spectacles; for after all it is the brain which really observes, and to which the eye and the microscope are alike secondary. Precisely as the most thorough anatomist might be utterly ignorant of physiology, so may the microscopist be also; the difference between the physiologist and the mere anatomist being that the one studies the working of the machine, the other its material structure only. Microscopy and the anatomy of gross forms are, however, equally valuable when subjected to the utilizing power—the intellect.

As a corollary to this proposition, we have to remark also that the observations made with this, as with any other optical instrument, are of limited force. Thus, the eye perceives a certain tint of skin in a sick man; this may be of great importance *in connection with other phenomena*, in diagnosis, but taken by itself it is inconclusive. So also the microscope reveals the visible peculiarities of the elements of a tumor, but these are nugatory when unconnected with the clinical history of the case. Hence we must be careful not to overstate our claim,—not to demand for the microscope the credit of powers which it does not and cannot possess.

Microscopic observations should always be made with the utmost care and thoroughness; generally several different portions of the tissue or morbid growth should be examined. Often it is of great use to study the elements not only *in situ*, as in a thin section, but also individually, by breaking them up with needles, or by using sufficient pressure to separate them. To speak here of the employment of reagents would be unnecessary. Perhaps it may also be thought superfluous for me to mention here the absolute necessity of extreme attention to the cleanliness of the glass slides, covers, and other accessories employed.

Accurate records should always be kept of our observations; and correct drawings of the objects viewed are of great value. By one or both of these means of *fixation*, if I may call them so, the experience gained is made available in the highest possible degree. When the elementary forms display a tendency to any particular mode of grouping, this should be carefully noted; and the same may be said of the blood-vessels and nerve-filaments sometimes observed.

If microscopists carry on their work not as mere microscopists, but with a just estimate of the proper value and office of the instrument, with a spirit of earnest and cautious inquiry, seeking only the elaboration of truth, they may confidently look for an ample reward of their efforts. For it is only by such as serve science in this way, that real advances are made; they alone carry out the precept laid down by the highest possible authority,—"Prove all things; hold fast that which is good."

[Mention should have been made, in connection with the general subject of the cell-doctrine, of a paper entitled "The Cell; its Physiology, Pathology and Philosophy," which was published by Dr. Waldo J. Burnett, of Boston, in the Transactions of the American Medical Association for the year 1853. This paper, based upon original observations, forms a valuable addition to micrographic literature.]

